# Reply to: Letter to the Editor on Endovascular Revascularization with Stent Implantation in Patients with Acute Mesenteric Ischemia Due to Acute Arterial Thrombosis: Clinical Outcome and Predictive Factors

**DOI:** 10.1007/s00270-021-02985-0

**Published:** 2021-10-26

**Authors:** Federico Pedersoli, Kai Schönau, Maximilian Schulze-Hagen, Sebastian Keil, Peter Isfort, Alexander Gombert, Patrick Hamid Alizai, Christiane K. Kuhl, Philipp Bruners, Markus Zimmermann

**Affiliations:** 1 Department of Diagnostic and Interventional Radiology, University Hospital RWTH Aachen, Pauwelsstraße 30, 52074 Aachen, Germany; 2 Department of Vascular Surgery, University Hospital RWTH Aachen, Pauwelsstraße 30, 52074 Aachen, Germany; 3 Department of General, Visceral and Transplant Surgery, University Hospital RWTH Aachen, Pauwelsstraße 30, 52074 Aachen, Germany

Dear Editor,

We thank Dr. Lauro and Prof. Sapienza for their thoughtful correspondence to our publication on endovascular revascularization in patients with acute mesenteric ischemia, where we report our experience on 40 consecutive patients who underwent stent implantation after acute mesenteric ischemia secondary to thrombosis of the proximal celiac or superior mesenteric artery [1]. We found that despite a high technical success rate (36/40 patients; 90%), the 30-day mortality rate was high (25/40 patients; 62.5%). Lauro et al. compared this with their own results, published on patients with mesenteric ischemia undergoing emergency surgery in an intestinal stroke center. Mortality in their patient cohort was lower than that observed in our cohort; 31 out of their total 63 patients deceased in the post-operative period (49.2%). The correspondents argue that the specific setting of an intestinal stroke center, where inter-disciplinary decisions are made faster, and/or the surgical approach might explain the lower mortality in their cohort [2].

However, as highlighted by the guidelines of the World Society of Emergency Surgery [3] and in the European Society for Trauma and Emergency Surgery (ESTES) [4], mesenteric ischemia due to arterial thrombosis, arterial embolism, venous thrombosis and non-occlusive disease can be considered as four different clinical entities, with different risk factors, clinical presentation and management. More important—and of special relevance to this correspondence–is the fact that they are associated with quite different prognosis, with arterial thrombosis having the worst prognosis compared to other causes of mesenteric ischemia [5]. It was these patient group that we specifically focused on in our manuscript; only patients with acute mesenteric ischemia resulting from arterial thrombosis at the ostia of the mesenteric vessels were included in our study, and we report on a follow-up of nine years (2011 – 2019). In the cohort published by Lauro et al., less than half of the patients (30/63) suffered from mesenteric ischemia caused by arterial thrombosis; the remaining had other types of mesenteric ischemia, including vasospasm and chronic ischemia. Accordingly, we believe that the differences in mortality observed in the two patient cohorts could also be explainable by the variable composition of the two cohorts. Moreover, the study published by the correspondents had a shorter follow-up.

In order to account for confounding variables and obtain clear actionable insight, a strict patient selection for studies evaluating the outcome of different treatment options for mesenteric ischemia is paramount to get clear evidence in future. In this context, establishing national or international patient registers may accomplish larger study populations and better data to improve the overall level of evidence regarding the treatment of patients with different causes of mesenteric ischemia.

We fully agree with the correspondent in that a seamless, fast inter-disciplinary treatment of patients with suspected or proven mesenteric ischemia is of key importance to improve outcome of these patients. Such inter-disciplinary decision making and treatment is certainly best achieved in dedicated intestinal stroke centers, but also in larger tertiary care hospitals where interventional radiologists offer the same 24/7 emergency treatment service as surgeons do.
